# A Narrative Review of HRV Metric Selection for Applied Monitoring: RMSSD as the Default Metric for Sport and Exercise

**DOI:** 10.3390/jfmk11030304

**Published:** 2026-07-31

**Authors:** Michael R. Esco

**Affiliations:** Department of Kinesiology, The University of Alabama, Tuscaloosa, AL 35487, USA; mresco@ua.edu

**Keywords:** HRV, RMSSD, athlete monitoring, autonomic regulation, recovery monitoring

## Abstract

Heart rate variability (HRV) monitoring is commonly used in sport and exercise science to assess recovery, training adaptation, and performance readiness. However, the routine reporting of numerous HRV metrics with substantial conceptual and physiological overlap has created inconsistency regarding which indices should be prioritized for applied monitoring. This narrative review synthesizes the contemporary literature to provide practical recommendations regarding HRV metric selection in applied monitoring, with particular emphasis on whether the root mean square of successive differences between adjacent normal-to-normal intervals (RMSSD) may serve as the primary metric for routine HRV monitoring in many healthy sport and exercise populations. RMSSD demonstrates several characteristics favorable for applied monitoring, including strong physiological relevance, favorable reliability, compatibility with ultra-short recording durations, relative robustness during spontaneous breathing, computational simplicity, and suitability for longitudinal monitoring frameworks. Although additional HRV metrics remain valuable for addressing specific physiological, mechanistic, or research questions, the current evidence supports RMSSD as the primary metric for routine HRV monitoring in many sport and exercise settings.

## 1. Introduction

Heart rate variability (HRV) has become one of the most commonly used tools for monitoring autonomic function, recovery status, and adaptation to training in sport and exercise settings [1,2]. Advances in wearable technology, smartphone applications, and commercially available software have made HRV assessment increasingly accessible [3]. Consequently, the use of HRV monitoring has expanded substantially over the past decade, particularly in the context of ultra-short-term recordings obtained under mobile conditions [4,5].

Despite the growing popularity of HRV monitoring, considerable inconsistency remains regarding the selection and interpretation of HRV metrics. The literature commonly discusses a variety of time-domain, frequency-domain, and non-linear indices, many of which demonstrate substantial mathematical and physiological overlap [6,7,8,9]. This abundance of HRV metrics creates uncertainty regarding which indices should be prioritized for routine monitoring or selected to address specific research questions. This issue is particularly relevant in applied settings where simplicity, reliability, and interpretability are essential for decision-making [1,10]. Among the numerous available HRV indices, RMSSD has emerged as one of the most commonly used metrics in sport and exercise science [1,10,11]. However, questions remain regarding whether RMSSD alone may be sufficient for routine short-term resting HRV monitoring, or whether the routine inclusion of numerous additional metrics meaningfully enhances interpretation and applied decision-making [1,8,9].

Previous reviews have primarily focused on the physiological underpinnings, measurement standards, and practical implementation of HRV [2,3,6,8,10,11]. For example, a recent narrative review synthesized recommendations for implementing and interpreting HRV-based monitoring in athletes, including recording procedures, measurement duration, and longitudinal interpretation [11]. However, comparatively less attention has been devoted to the more fundamental question of which HRV metric should serve as the primary outcome measure for routine short-term resting assessments in sport and exercise settings.

The purpose of this narrative review was to provide practical methodological recommendations for HRV metric selection in applied monitoring, with particular emphasis on whether RMSSD may serve as the default metric for routine short-term resting assessments performed in sport and exercise settings. Specifically, this review argues that RMSSD may be sufficient as the primary HRV metric for most short-term resting assessments in these settings while also identifying circumstances in which additional HRV metrics may provide complementary information. Accordingly, the recommendations presented in this review are intended for healthy, physically active adults and should not be generalized to children, clinical populations, individuals with cardiac arrhythmias, patients receiving medications known to alter autonomic regulation (e.g., beta-blockers), or other specialized populations without population-specific evidence.

## 2. Methods

This manuscript was conducted as a narrative review, which was intended to synthesize the contemporary literature and provide practical methodological recommendations regarding HRV metric selection for short-term resting assessments in sport and exercise settings. Unlike a systematic review, the objective was not to identify every available publication related to a specific research question, but rather to critically evaluate foundational and contemporary evidence relevant to the practical selection and interpretation of HRV metrics.

Literature searches were conducted from November 2025 through February 2026 using the PubMed, Scopus, and Google Scholar databases. Searches used combinations of terms including “heart rate variability”, “HRV”, “RMSSD”, “SDNN”, “frequency-domain”, “nonlinear HRV”, “athlete monitoring”, “recovery”, “ultra-short-term HRV”, “autonomic regulation”, and “training adaptation”. Additional articles identified through the reference lists of retrieved publications and existing reviews were also considered when relevant. Priority was given to consensus statements, systematic and narrative reviews, and original investigations that informed the physiological interpretation, reliability, validity, and practical application of HRV metrics for short-term resting assessments in sport and exercise settings. Papers that fell outside the intended scope of this narrative review, such as those that focused on clinical or pediatric populations, or on topics not directly informing HRV metric selection for applied monitoring, were generally not included. Although the primary emphasis was placed on recent literature, seminal publications that established fundamental concepts in HRV measurement and interpretation were included when necessary to provide historical and methodological context.

## 3. The Problem: Metric Proliferation

As HRV monitoring has increased in popularity, so too has the number of reported HRV indices within the literature. Contemporary studies routinely report multiple time-domain, frequency-domain, and non-linear parameters from a single heartbeat interval recording [8,12]. Common metrics include SDNN, RMSSD, pNN50, high-frequency power (HF), low-frequency power (LF), LF/HF ratio, SD1 and SD2 from Poincare plotting, sample entropy, and detrended fluctuation analysis indices [6,8,13,14]. Although each metric is often presented as providing distinct physiological information, substantial overlap exists among many commonly reported indices, and several are not suitable during short-term recordings [8,9,15,16].

For example, RMSSD and SD1 have been shown to exhibit a perfect linear relationship (Pearson r = 1.00) [9]. Furthermore, RMSSD demonstrates very strong associations with HF power [8], with reported correlations ranging from r = 0.94 to 0.97 under resting and immediate post-exercise conditions [17]. Likewise, SDNN has been shown to be strongly associated with LF power, with correlation coefficients ranging from r = 0.90 to r = 0.94 across resting and immediately post-exercise conditions [17]. Consequently, ratios such as SDNN/RMSSD appear to provide interpretations conceptually similar to those often inferred from LF/HF [8,9,17,18,19]. Similar overlap exists among numerous additional HRV indices because many are ultimately derived from the same underlying heartbeat interval fluctuations, with some measures demonstrating near-perfect associations under resting and immediate post-exercise conditions [8,9,17,18,19]. Consequently, metrics originating from different analytical domains are frequently interpreted as distinct physiological constructs despite sharing considerable mathematical and physiological commonality [19]. The routine reporting of numerous highly related indices may therefore contribute more to redundancy than to meaningful physiological insight.

This issue is further complicated by longstanding disagreement regarding the physiological interpretation of several HRV indices [6,7,8,9]. Frequency-domain measures, particularly LF power and the LF/HF ratio, have historically been interpreted as markers of sympathetic activity or sympathovagal balance [6,13]. However, these interpretations have been increasingly challenged, with evidence suggesting that such measures are influenced by multiple physiological processes and may not provide clear or isolated representations of sympathetic modulation [7,20,21,22].

The widespread availability of automated HRV software may have unintentionally contributed to this problem. Modern platforms can generate dozens of HRV metrics instantaneously, encouraging comprehensive reporting despite limited evidence that additional parameters improve practical decision-making or research findings [3,10,11,23]. Although the inclusion of multiple HRV metrics may occasionally provide additional physiological context, excessive reporting of highly related indices may increase analytical flexibility without necessarily improving interpretation [7,10,24]. Because many HRV parameters reflect overlapping mathematical and physiological properties, the routine inclusion of numerous metrics may complicate interpretation more than it enhances practical decision-making or scientific understanding [8,9,10,19]. In many applied settings, a single well-selected HRV metric may be sufficient for monitoring athlete recovery status or training adaptation, and the inclusion of additional metrics may not substantially alter practical interpretation or decision-making [10,11]. The practical characteristics, strengths, limitations, and recommended applications of these commonly reported HRV metrics are summarized in Table 1.

Ultimately, the goal of applied research and practice is not to maximize the number of reported variables, but rather to identify metrics that are reliable, interpretable, and practically useful for longitudinal assessment [1,10,11,25,26]. Therefore, an important question emerges: if a single metric consistently captures the majority of relevant information obtained from short-term HRV recordings, is the routine reporting of numerous additional indices necessary? For many common applied monitoring scenarios, the current evidence suggests that the answer is often “no” [1,10,11].

## 4. RMSSD as the Default HRV Metric for Practical Monitoring

Importantly, all HRV metrics represent indirect mathematical representations of underlying physiological processes rather than direct measurements of autonomic activity itself [6,7,8,13]. Therefore, no single HRV parameter fully captures the complexity of autonomic regulation [6]. However, the usefulness of an HRV metric should not be determined solely by the extent to which it reflects a single physiological mechanism [1,10,11]. Rather, the selection of HRV metrics should ideally be guided by the specific objective of the assessment and a clearly defined physiological or research rationale. Factors such as reliability, interpretability, and suitability for the intended application are also important considerations, particularly for field-based monitoring [1,10,11]. Based on the current evidence, RMSSD possesses many of these characteristics, and as such, appears well suited for applied research and routine monitoring in sport and exercise settings [11].

An important advantage of RMSSD is its favorable reliability characteristics relative to many other commonly reported HRV indices [16,27]. Compared to several frequency-domain and non-linear parameters, RMSSD generally demonstrates lower day-to-day variability and more consistent behavior during repeated resting assessments [16,27,28]. For example, Besson et al. [28] reported intraclass correlation coefficients exceeding 0.75 across five consecutive days, with a standard error of measurement of 5.3 ms for resting RMSSD. These characteristics make it especially well-suited for athlete monitoring over long-term training regimens [1,10,11]. The logarithmic transformation of RMSSD (LnRMSSD) may further improve statistical properties by reducing skewness and minimizing the influence of outliers within the recording, thereby enhancing reliability during longitudinal interpretation [6,29,30]. Because of these favorable reliability characteristics, RMSSD has also been shown to perform well during ultra-short recordings. Several investigations have reported strong agreement between 1 min RMSSD values and criterion-length (i.e., 5 min) recordings [5,15,16,25,31]. This relationship has been quantified by intraclass correlation coefficients exceeding 0.95 under resting and immediate post-exercise conditions [5,16]. However, these findings should not be generalized to non-steady-state conditions characterized by rapidly changing autonomic activity or persistently elevated heart rate, where longer recording durations may be more appropriate.

This characteristic is particularly important for field-based application and longitudinal monitoring protocols where daily assessment and athlete compliance is essential [10,11]. Numerous studies have demonstrated the value of repeated daily HRV assessment, rather than isolated measurements, for monitoring recovery status and training adaptation [2,32,33,34,35,36]. Frequent recordings are also necessary for calculating weekly averages and coefficients of variation derived from daily RMSSD measurements, which may provide insight into long-term adaptation and short-term recovery dynamics, respectively [11]. Therefore, RMSSD’s ability to be accurately obtained from ultra-short recordings as short as 1 min further supports its suitability for routine longitudinal monitoring in applied sport and exercise settings [1,10,11].

An additional advantage of RMSSD is its suitability for distinguishing meaningful physiological changes from normal biological variability during longitudinal monitoring [1,10]. In applied settings, daily fluctuations in HRV may occur due to numerous factors unrelated to training adaptation alone, including sleep, hydration, psychological stress, and normal measurement variability [1,8,12]. Consequently, an effective monitoring metric should demonstrate sufficient stability and reliability to allow meaningful physiological signals to emerge over time [37]. The favorable reliability characteristics of RMSSD, particularly when interpreted using repeated measurements, weekly averages, and coefficients of variation, may improve the ability to detect meaningful changes in training status while minimizing interpretive noise [11,32,33,34,35,36].

Another practical advantage of RMSSD is its relative robustness during spontaneous breathing conditions commonly encountered in applied monitoring environments [38]. Although respiration influences all HRV measures to some extent, several HRV indices, particularly frequency-domain parameters, may be especially sensitive to alterations in breathing frequency and respiratory pattern [39,40,41,42,43]. In contrast, RMSSD appears comparatively less influenced by uncontrolled respiratory variation during short-term resting assessments, which may enhance interpretability under field-based conditions [10,38]. This characteristic may improve practicality when testing large groups of athletes or implementing unsupervised daily monitoring protocols [11].

In addition, RMSSD is computationally simple and widely accessible [6,8]. Unlike several frequency-domain and non-linear approaches that require more advanced signal processing procedures, RMSSD can be readily calculated from exported R-R interval data using relatively simple spreadsheet-based computations [8,10,28]. Modern wearable devices, smartphone applications, and commercially available HRV software platforms also routinely provide RMSSD-derived values, facilitating standardized implementation across laboratories and field settings [3,4,28]. This widespread adoption has contributed to substantial normative and comparative literature, further strengthening its practical utility [2,8,44]. Mobile HRV applications and wearables have further increased accessibility by allowing rapid daily RMSSD monitoring using convenient field-based procedures [3,11].

Because of these features, RMSSD integrates well within contemporary approaches to longitudinal athlete monitoring [1,10,11]. Numerous investigations have demonstrated that RMSSD responds sensitively to training-related stress and recovery [1,2,45]. For example, reductions in RMSSD have been observed during intensified training periods and states of accumulated fatigue, whereas restoration or increases in RMSSD have often accompanied improved recovery and positive adaptation to training [2,33,34,35,46]. Additionally, weekly averages and coefficients of variation in RMSSD have demonstrated utility for improving longitudinal interpretation by reducing the influence of normal day-to-day fluctuations while enhancing sensitivity to meaningful training-related changes [33,34,47,48]. Consequently, RMSSD appears to provide useful insight into day-to-day physiological readiness and recovery status, and long-term adaptation to training [1,10,11].

The practical value of RMSSD extends beyond the metric itself and depends heavily on the consistency with which measurements are obtained and interpreted. Because HRV is influenced by numerous behavioral, environmental, and physiological factors, meaningful longitudinal monitoring requires standardized measurement procedures that minimize unnecessary sources of variability [1,11,12]. Consequently, practitioners should strive to maintain consistent testing conditions whenever possible, including time of day, body position, recording duration, device, and measurement environment. Morning assessments performed shortly after waking and before food intake, caffeine consumption, or exercise have become common practice because they help reduce biological and behavioral variability while improving the comparability of repeated measurements [1,11].

Equally important is the recognition that isolated RMSSD values should generally be interpreted with caution. Day-to-day fluctuations in HRV may reflect normal biological variation or transient influences such as sleep quality, psychological stress, hydration status, illness, travel, or recent training load rather than meaningful changes in physiological readiness or adaptation [1,2,12]. Consequently, contemporary athlete-monitoring frameworks increasingly emphasize the interpretation of repeated measurements rather than individual observations. Weekly averages of daily RMSSD recordings may provide a more stable representation of autonomic status, whereas coefficients of variation may offer additional insight into the consistency of autonomic regulation over time [11,33,34,35,36,47]. These approaches reduce the influence of normal biological variability and may improve the detection of meaningful changes associated with training adaptation, accumulated fatigue, or recovery.

Practitioners should also recognize that HRV represents only one component of a comprehensive monitoring strategy. Although RMSSD provides valuable information regarding cardiac interval variability and recovery status, interpretation should ideally occur alongside other indicators of athlete health and performance, including subjective wellness, training load, sleep, performance testing, and relevant physiological measures when available [1,2,10,11]. Integrating RMSSD within a broader monitoring framework may provide greater practical value than relying on any single metric in isolation. Accordingly, the greatest strength of RMSSD may lie not simply in its physiological associations, but in its ability to provide a reliable, repeatable, and practical signal that contributes to informed longitudinal decision-making.

## 5. Contextualizing RMSSD Interpretation

Beyond the practical advantages, RMSSD is widely interpreted as a parasympathetically influenced HRV index [6,8]. This interpretation is primarily derived from the physiological characteristics of autonomic control over the sinoatrial node [6,8]. Parasympathetic influences occur rapidly and can alter cardiac cycle length on a beat-to-beat basis, whereas sympathetic influences generally occur more slowly over several seconds [49,50,51]. Because RMSSD is derived from successive differences between adjacent R-R intervals, it preferentially captures rapid short-term fluctuations in cardiac cycle length [6,8]. Consequently, RMSSD closely parallels respiratory sinus arrhythmia and often demonstrates strong associations with HF power [8,9,52]. RMSSD has also shown behavior consistent with expected parasympathetic withdrawal during exercise and parasympathetic reactivation during recovery [45,51]. Experimental evidence further supports parasympathetic involvement, as pharmacological parasympathetic blockade using atropine substantially attenuates RMSSD, whereas sympathetic blockade produces smaller effects under resting conditions [53,54].

However, despite these observations, the relationship between RMSSD and parasympathetic activity is likely more nuanced than a simple one-to-one physiological correspondence. RMSSD is fundamentally a mathematical description of short-term cardiac interval variability rather than a direct measurement of vagal nerve firing or parasympathetic outflow [8,55,56]. Although parasympathetic modulation appears to contribute substantially to RMSSD, the sinoatrial node is also influenced by a variety of additional factors, such as respiratory mechanics, baroreflex activity, intrinsic pacemaker dynamics, hormonal influences, and thermoregulation [57]. Consequently, physiological association should not be interpreted as physiological specificity [54].

This distinction is important because RMSSD is sometimes interpreted as directly reflecting parasympathetic modulation. Therefore, rather than using statements that imply RMSSD is a direct vagal or parasympathetic marker, researchers may wish to use more physiologically conservative language when describing HRV findings, particularly in study titles, aims, and conclusions. For example, phrases such as “RMSSD responses to training” or “parasympathetically associated HRV indicators” may be more physiologically conservative than definitive statements regarding direct vagal activity. Nevertheless, the value of RMSSD within sport and exercise settings extends beyond physiological specificity. Instead, its utility for applied monitoring derives from its practical convenience, responsiveness to training-related stress and recovery, and suitability for repeated longitudinal assessment [1,2,10,11].

Additionally, the practical utility of RMSSD should not be interpreted to mean that it fully captures all dimensions of autonomic regulation or cardiovascular complexity. Because RMSSD primarily reflects rapid short-term fluctuations in cardiac interval variability, it may provide less insight into slower oscillatory patterns, sympathetic-related autonomic dynamics, or complex non-linear characteristics of heart rate behavior [6,7,8,14]. Consequently, alternative HRV metrics may still provide meaningful physiological information in specific contexts, such as mechanistic laboratory investigations, controlled respiration protocols, prolonged electrocardiographic recordings, studies focused on autonomic dysfunction, or investigations specifically examining fractal and complexity-related properties of HRV dynamics [6,14,18,22].

## 6. When Are Additional HRV Metrics Justified?

Although RMSSD serves as the default HRV metric for many short-term resting applications, additional HRV metrics remain valuable when the objective extends beyond routine longitudinal monitoring. Accordingly, the selection of complementary HRV indices should be guided by a specific physiological, methodological, or research rationale rather than incorporated routinely into every monitoring protocol [11,54]. Several published frameworks illustrate circumstances in which a multi-metric approach may provide additional physiological insight.

Perhaps the strongest published argument for routine multi-metric HRV assessment has been advanced by Schmitt et al. [29], who proposed that HRV may be useful for differentiating fatigue phenotypes associated with functional overreaching, non-functional overreaching, and overtraining syndrome. According to this framework, RMSSD (or LnRMSSD) provides a sensitive indicator of global parasympathetic modulation but may not adequately distinguish among distinct autonomic response patterns [29]. By combining time-domain and frequency-domain indices obtained in both supine and standing positions, it may be possible to identify physiological profiles that provide greater insight into the nature of accumulated fatigue than RMSSD alone [29]. This approach is particularly attractive when the objective extends beyond routine readiness monitoring to the characterization of maladaptive training responses [1,11,29]. However, its implementation requires more complex testing procedures, greater analytical expertise, and less feasible field-based testing protocols [10,11,29]. Therefore, while multi-metric assessment may be justified when the objective is to characterize complex fatigue states or suspected maladaptation, its broader application to routine monitoring in exercise and sport contexts remains uncertain [10,11,29].

Beyond fatigue phenotyping, additional HRV metrics may also be selected when they directly address a specific physiological or mechanistic research question. For example, frequency-domain measures may be appropriate when spectral characteristics are themselves central to a research question. HF power may provide useful information during mechanistic investigations of respiratory-related cardiac variability, particularly when breathing frequency and respiratory pattern are controlled [6,40,41]. Likewise, LF-derived measures may be considered when research questions specifically involve baroreflex-related or slower oscillatory processes [20,21,22]. Similarly, non-linear HRV metrics, including Poincaré plot-derived indices, entropy measures, and fractal analyses, may provide valuable insight into complex physiological dynamics and autonomic system behavior [8,14]. However, these approaches remain less standardized for routine field-based assessment and may therefore present greater challenges for interpretation and implementation than established time-domain measures [10,14,18]. Therefore, unless these metrics address a specific physiological or mechanistic question, their routine inclusion in applied sport and exercise monitoring is unlikely to substantially improve interpretation beyond that provided by RMSSD.

Among the more established time-domain alternatives, SDNN may be particularly useful as a complementary metric because it represents the overall variability of normal-to-normal intervals throughout the recording period [6]. While RMSSD preferentially reflects rapid beat-to-beat fluctuations, SDNN incorporates both rapid and slower components of cardiac interval variability [6,9,54]. This broader representation may be especially informative during assessments intended to characterize variability beyond predominantly short-term fluctuations [6,8]. However, during short-term resting recordings, SDNN should generally be interpreted alongside RMSSD rather than as a replacement for it [17,18].

Recent investigations have explored whether RMSSD and SDNN can be combined to produce simplified time-domain indicators of broader autonomic behavior. One approach uses the SDNN/RMSSD ratio as a simplified time-domain alternative to relationships traditionally examined using LF/HF. Ultra-short recordings of the SDNN/RMSSD ratio have demonstrated very large associations with LF/HF (r = 0.72–0.89) derived from conventional 5 min recordings at rest and immediately following maximal exercise [17]. This approach may be attractive in applied settings because it can be calculated from two familiar time-domain metrics without requiring frequency-domain analysis. However, the SDNN/RMSSD ratio should not be interpreted as a direct measure of sympathetic activity or sympathovagal balance [7,20,21,22,43], and its practical value beyond the individual RMSSD and SDNN values remains to be established.

Other emerging approaches use RMSSD and SDNN to derive modified versions of metrics originally calculated from Poincaré plot analysis. These include a modified stress score derived from SDNN and a parasympathetic:sympathetic ratio incorporating RMSSD and the modified stress score [18,58,59]. Because these measures can be calculated from ultra-short recordings, they may provide a computationally simple approach for examining the relationship between short-term and broader cardiac interval variability in field-based settings. Preliminary findings suggest that these indices can be obtained reliably from brief recordings and may respond to acute physiological perturbations [18,58]. Therefore, these derived measures may be appropriate when practitioners wish to complement RMSSD with a broader or sympathetic-related perspective that RMSSD alone is not intended to provide. Nevertheless, they remain relatively novel, and the current evidence is insufficient to determine whether they meaningfully improve athlete monitoring, recovery assessment, or training-related decision-making beyond RMSSD and SDNN interpreted separately.

Collectively, these alternative approaches illustrate an important direction for future HRV research. Rather than continuing to generate additional indices solely because they are mathematically available, future investigations should determine whether new metrics meaningfully improve interpretation or decision-making beyond RMSSD. Outcomes should include not only statistical associations but also improvements in training prescription, recovery assessment, readiness classification, and longitudinal decision-making.

**Figure 1:**
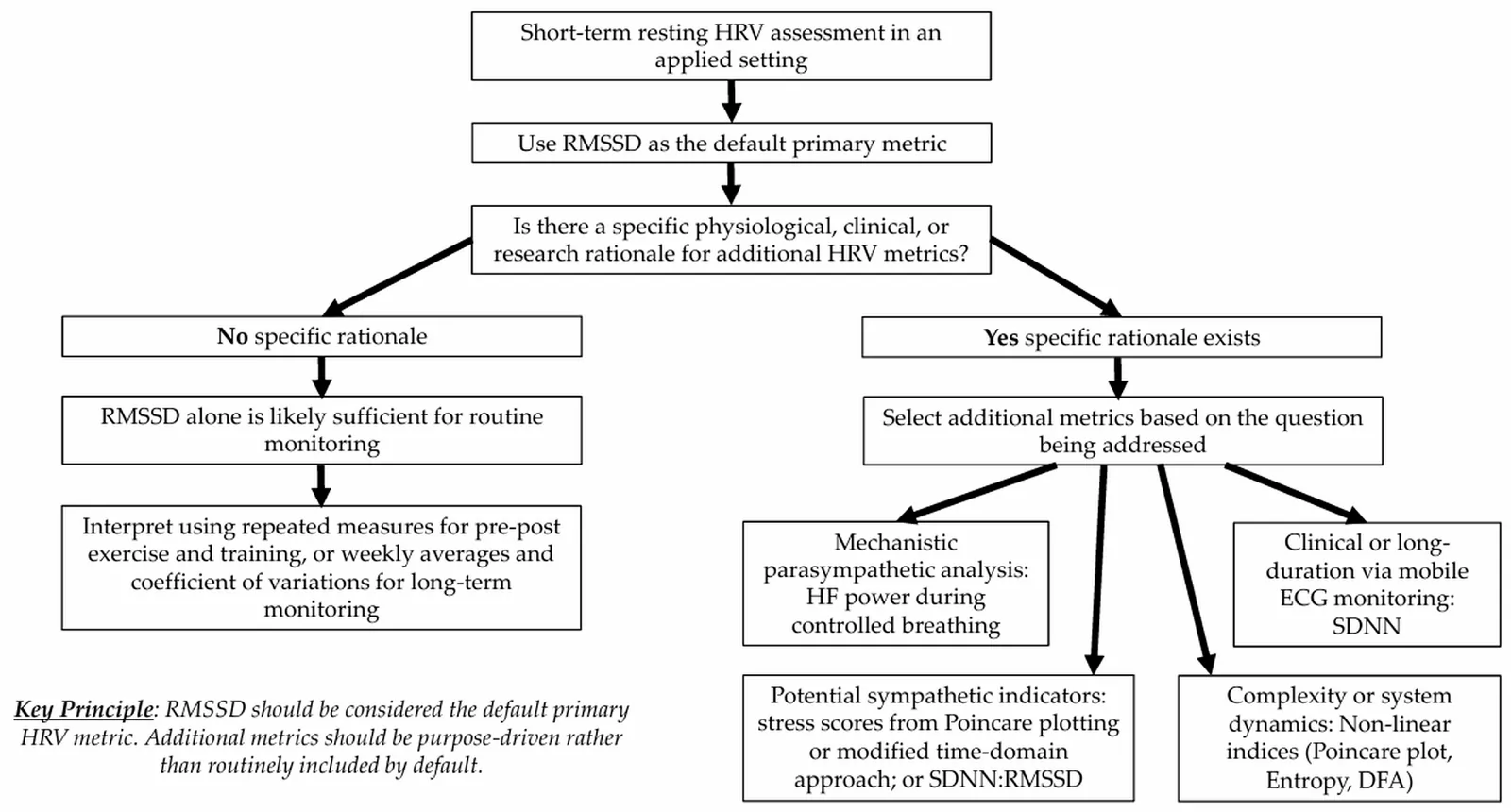
Proposed conceptual framework for HRV metric selection in applied monitoring. The framework is based on the literature synthesized in this review and is intended as a practical heuristic rather than a formally validated decision-making tool.

The proposed framework for HRV metric selection may be especially relevant in applied sport and exercise settings, where the primary objective is often to monitor training adaptation, recovery status, or readiness rather than to comprehensively characterize autonomic physiology. In such settings, the most useful HRV metric may not necessarily be the one that reflects the greatest number of physiological mechanisms, but rather the one that provides the most stable, interpretable, and practically actionable information [1,2,11,28,30]. Accordingly, regardless of whether a single metric or multi-metric approach is adopted, HRV metric selection should be guided by the purpose of the assessment rather than by convention or automated software output alone. Figure 1 presents a proposed framework for selecting HRV metrics according to the objectives of the assessment.

In practice, the proposed framework is intended to guide metric selection before HRV data are collected rather than during interpretation. For example, a practitioner performing routine daily readiness or recovery monitoring would typically select RMSSD as the primary metric because it provides a robust, well-validated indicator of short-term parasympathetic-related variability. If the monitoring objective extends beyond routine readiness assessment to address a specific physiological question, such as examining overall HRV or obtaining a complementary sympathetic-related perspective, additional metrics such as SDNN or derived indices including the modified stress score may be incorporated when supported by an appropriate rationale. Conversely, mechanistic investigations of autonomic regulation may justify the inclusion of frequency-domain or non-linear metrics because these approaches address physiological questions that RMSSD alone is not intended to answer. Thus, the framework encourages practitioners to begin with the objective of the assessment and select only those HRV metrics that provide information relevant to that objective.

## 7. Conclusions

The current evidence supports RMSSD as the most appropriate default HRV metric for short-term resting assessments performed in applied sport and exercise settings. Although RMSSD does not fully characterize the complexity of autonomic regulation, its favorable reliability, responsiveness to training-related stress and recovery, compatibility with ultra-short recordings, computational simplicity, and suitability for longitudinal monitoring collectively support its routine implementation within applied monitoring frameworks [11,46,60,61,62]. However, RMSSD should not be interpreted as a direct measure of parasympathetic activity. Instead, its value for applied sport and exercise monitoring lies not in physiological specificity, but in its ability to provide a reliable, interpretable, and practically useful indicator of short-term cardiac interval variability for longitudinal assessment. Likewise, recommending RMSSD as the default metric for applied settings does not diminish the physiological or research value of alternative HRV metrics. Rather, additional indices are likely to provide the greatest benefit when they meaningfully improve interpretation beyond RMSSD or address specific mechanistic questions.

This review has several limitations that should be acknowledged. As a narrative review, it did not employ a systematic literature search or formal study selection procedures, and the recommendations therefore reflect the author’s synthesis and interpretation of the available evidence. In addition, the discussion primarily focuses on short-term resting HRV assessment in applied sport and exercise settings and may not directly extend to clinical populations, or other specialized applications of HRV analysis.

Several important opportunities remain for future research. Investigations should determine whether emerging metrics, including the SDNN/RMSSD ratio and modified stress score, provide meaningful physiological or practical value beyond RMSSD alone. Additional work is also needed to establish standardized interpretation frameworks for complementary HRV metrics, evaluate their performance across diverse populations and monitoring conditions, determine whether purpose-driven HRV metric selection improves longitudinal decision-making in applied settings, and examine how emerging analytical approaches, including machine learning, may further refine HRV interpretation.

Ultimately, the objective of HRV monitoring is not to maximize the number of reported variables but to obtain reliable, interpretable information that meaningfully informs practice. When the purpose of the assessment is clearly defined, HRV metric selection becomes more straightforward. For most short-term resting applications in sport and exercise settings, RMSSD provides an appropriate default measure, while additional HRV metrics should be incorporated only when they address specific physiological or research questions that RMSSD alone is unlikely to answer.

## Figures and Tables

**Table 1:** Practical Considerations for Common HRV Metrics in Applied Monitoring.

Metric (Domain)	Interpretation	Strengths	Limitations	Recommended Use
RMSSD (Time)	Short-term cardiac interval variability strongly associated with parasympathetic modulation	Strong reliability; compatible with ultra-short recordings; computationally simple; robust during spontaneous breathing; well suited for long-term monitoring	Does not fully capture overall autonomic complexity or broader variability patterns	Primary metric for routine short-term resting HRV monitoring in sport and exercise settings
SDNN (Time)	Overall variability of normal-to-normal intervals	Provides broader representation of HRV; useful in long-duration recordings	Less physiologically specific during short-term recordings; influenced by slower oscillations	Complementary metric alongside RMSSD
pNN50 (Time)	Short-term cardiac interval variability strongly associated with parasympathetic modulation	Intuitive percentage-based interpretation	Sensitive to recording artifacts and often redundant with RMSSD	Limited additional value beyond RMSSD during routine short-term monitoring
HF Power (Frequency)	Respiratory-related variability commonly associated with parasympathetic modulation	Strong physiological rationale under controlled conditions	Influenced by breathing frequency and respiratory pattern; requires spectral analysis	Laboratory-based investigations with controlled respiration
LF Power (Frequency)	Historically interpreted as sympathetic modulation or baroreflex-related activity	May provide information regarding broader autonomic oscillations	Physiological interpretation remains controversial	Use cautiously and only with a clearly defined rationale
LF/HF (Frequency)	Historically interpreted as sympathovagal balance	Commonly reported in historical literature	Increasingly criticized for oversimplified physiological interpretation	Generally not recommended for routine applied monitoring
SD1 (Non-linear)	Short-term variability from Poincaré plot analysis	Visually intuitive; mathematically equivalent to RMSSD	Provides little additional information beyond RMSSD	Alternative visualization approach rather than a necessary additional metric
SD2 (Non-linear)	Longer-term variability from Poincaré plot analysis	May provide broader variability context	Interpretation less established during short-term recordings	Potential complementary metric when combined with RMSSD
Entropy/DFA (Non-linear)	Complexity and fractal characteristics of HRV dynamics	May provide insight into autonomic system complexity	More difficult interpretation; less standardized for field use	Advanced mechanistic or research-focused applications

RMSSD, root mean square of successive differences; SDNN, standard deviation of normal-to-normal intervals; pNN50, percentage of adjacent normal-to-normal intervals differing by more than 50 ms; HF, high-frequency power; LF, low-frequency power; LF/HF, LF-to-HF ratio; SD1 and SD2, Poincaré plot-derived indices; DFA, detrended fluctuation analysis. References supporting the summary of each HRV metric within the Table are as follows: RMSSD [1,6,10,11]; SDNN [6,8,15,16]; pNN50 [6,8]; HF [6,7,8]; LF [6,7,20,21,22]; LF/HF [6,7,20,21,22]; SD1 and SD2 [8,13,14]; DFA [8,13,14,19].

## Data Availability

No new data were created or analyzed in this study. Data sharing is not applicable to this article.

## Abbreviations

The following abbreviations are used in this manuscript:

HF	High-frequency power
HRV	Heart rate variability
LF	Low-frequency power
LF:HF	Low-frequency to high frequency ratio
LnRMSSD	Natural logarithm of the root mean square of successive differences between adjacent normal-to-normal intervals
RMSSD	Root mean square of successive differences between adjacent normal-to-normal intervals
SD1	Standard deviation 1 from Poincaré plot analysis
SD2	Standard deviation 2 from Poincaré plot analysis
SDNN	Standard deviation of normal-to-normal intervals
SDNN:RMSSD	SDNN to RMSSD ratio
